# Developing an Oral Care Bundle to Reduce the Incidence of Mucosal Barrier Injuries in Hematology/Oncology Patients

**DOI:** 10.1097/pq9.0000000000000758

**Published:** 2024-09-25

**Authors:** Christian A. Schneider, Jennifer A. McDonnell, Erin K. O’Neill

**Affiliations:** From the Children’s Mercy - Kansas City, KS City, Mo.

## Introduction:

**Fig. 1.**
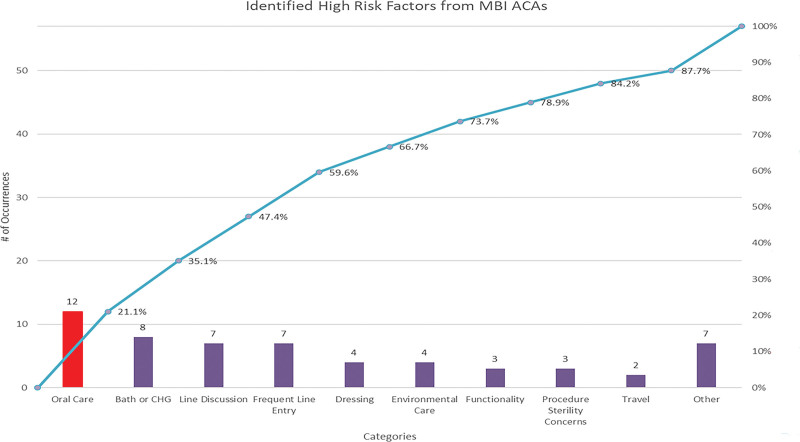
A pareto chart of risk factors identified from apparent cause analysis.

A mucosal barrier injury CLABSI (MBI-LCBI) occurs during periods of prolonged neutropenia in patients receiving cytotoxic chemotherapy for hematologic malignancies. Specials cause variation was identified in March 2023 (6.051/1,000 line days) and April 2023 (6.015/1,000 line days). A review of Apparent Cause Analysis’ identified poor compliance with oral care, specifically oral rinse being the biggest area of opportunity (Fig. 1).

## Objectives:

By the end of Fiscal Year (FY) 24, we aim to decrease our total MBI-LCBIs on the Hematology/Oncology/bone marrow transplant Unit from 16 to 10. The improvement team plans to develop an advanced oral care bundle targeted at patients with acute myeloid leukemia and patients admitted for bone marrow transplant.

## Methods:

**Fig. 2.**
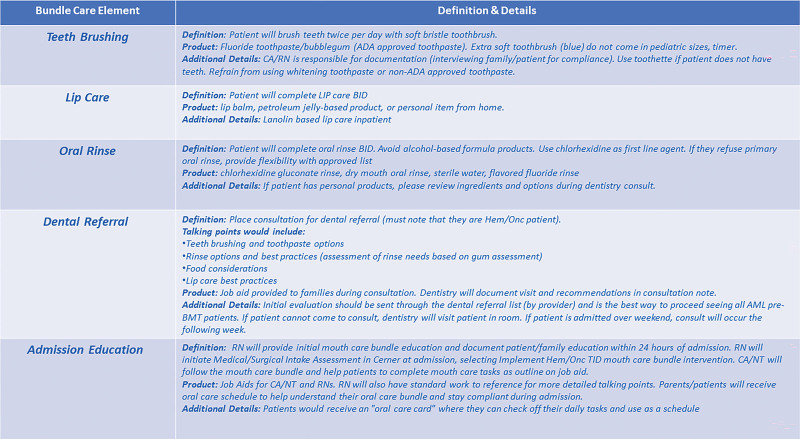
Revised oral care bundle.

An improvement team of infection preventionists, quality improvement specialists, nursing staff, physicians, and dentistry participated in an oral care bundle workshop. The 2-day workshop included the following:

- Review the current oral care bundle.
- Develop a revised oral care bundle that includes standards for teeth brushing, oral care, and lip care (Fig. 2).
- Explore new products for use.
- Consider new and innovative practices.
- Standardize education for patients and families.

The following timeline reflects the interventions for this project.

- January 2024: Implement new teeth brushing, lip care, oral rinse standards, and patient flyers.
- February 2024: Modification completed of admission and oral care documentation.
- May 2024: Human Factors provided recommendations to improve documentation.

## Results:

FY23 had 16 events with an MBI-LCBI rate of 2.217/1,000 line days. The committee implemented the oral care bundle in January 2024. FY24 has 12 events with an MBI-LCBI rate of 1.36/1,000 line days. The Hematology/Oncology/bone marrow transplant Unit has gone 86 days since the last MBI-LCBI in early May 2024. Oral rinse compliance improved when options were expanded to include a bubblegum-flavored option, a choice that was well-received by patients. Admission education and dental referral continue to be our areas of opportunity with adherence to the bundle. To address admission education compliance, the unit educator collaborated with Human Factors Scientists to improve the documentation workflow.

## Conclusion:

Engaging local leaders when conducting data analysis is essential, especially when optimizing buy-in and collaboration for improvement work. During the 2-day workshop, developing expectations and “rules” for brainstorming helped the improvement team achieve deliverables and minimize groupthink. The dental clinic was integral part of the improvement work and provided a unique lens, offering insights when brainstorming ideas for the oral care bundle. Leveraging the voice of the customer surfaced issues with the prior oral rinse, and this patient-centered approach improved compliance with the oral care bundle. The Patient and Family Advisory Council provided valuable feedback on patient education handouts and promoted health literacy.

